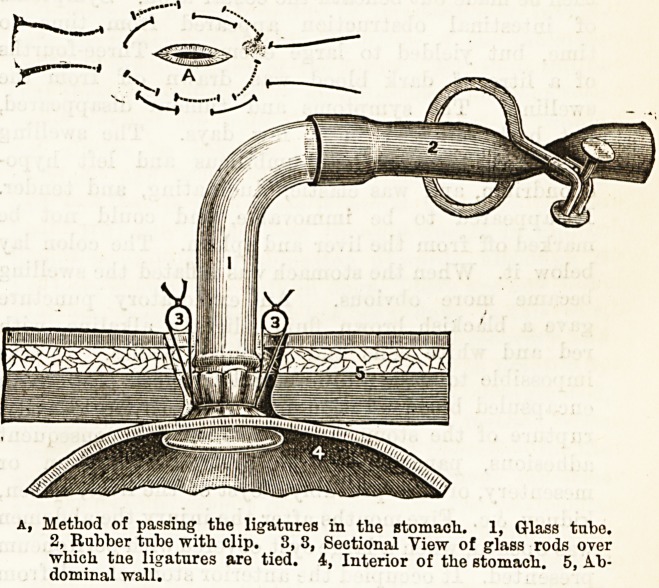# Surgery of the Œsophagus and Stomach

**Published:** 1894-06-02

**Authors:** 


### PROGRESS IN SURGERY.

### SURGERY OF THE ŒSOPHAGUS AND STOMACH.

**Œsophageal Stricture.**—Dr. Robert Abbe[1] reports a second case of *impermeable* stricture cured by cutting with a string. The patient—a child of three years—had swallowed half a cupful of caustic alkali six months before. Emaciation was extreme, and even milk could not be swallowed. Being unable to pass the smallest œsophageal bougie he performed gastrostomy, and from below was able to pass upwards a fine filiform bougie, bringing with it a string up into the mouth. He then succeeded with the aid of this string in passing very small conical bougies, dilating the stricture as much as it allowed, which was only a small catheter size. He then attached a string to a bougie, brought it out through the mouth, and with it performed œsophagotomy by see-sawing it up and down, while a larger conical bougie was pressed hard into the stricture from the stomach, thus cutting the strictured portion until the size had been reached which he thought the child's œsophagus would stand with safety. Only a few drops of blood were lost. The gastrostomy wound was closed and dilatation afterwards kept up by bougies. The child became strong and healthy. This method is useful for tight traumatic strictures low down in the gullet. Kendal Franks[2] also records a case of stricture of the œsophagus treated by dilatation from below, in a manner similar to that above recorded, but a plug of gauze with a silk ligature at either end was drawn backwards and forwards through the stricture, and was finally left in the stricture, the lower ligature being cut off. The stomach was immediately closed, and the plug was withdrawn in six hours.

Emil Mayer[3] reports a rare case of *congenital stricture* of the œsophagus treated by bougies. The patient, who was nine years of age, had lived on an entirely liquid diet. Drs. Stockton and Roswell Park relate[4] a case of diverticulum of the œsophagus, for which gastrostomy was performed. Eighteen months afterwards the patient continued to be well.

**Gastrostomy.**—Keen reports[5] an interesting case by *Witzel's method*, which he believes to be the best now known. The patient suffered from malignant stricture of the œsophagus. The operation proved entirely satisfactory; the tube was retained with no leakage, the wound healed perfectly, and the patient gained rapidly in strength, which continued. The patient was doing well, and there had been no leakage three months after operation. F. M. Caird also speaks[6] highly of this method of performing gastrostomy, and quotes two cases.

Plénières, in a new method of gastrostomy proposed by him, adopts somewhat similar principles to those of Witzel. In a series of experiments he deduced the fact that if the wall of the stomach is sewn by stitches, including all the coats except the mucous, into the abdominal wound, there will follow a contraction of the tissues, and a fold of mucous membrane will be formed corresponding to the external wound on the inner surface of the stomach. This he utilizes as a valve, making his opening after it has been formed, and between its folds he inserts a drainage or feeding tube, which is left in position for some days, and through it the patient is fed. After a time it can be removed, when the mucous folds will act as a valve and prevent the outflow of food. He quotes a case which he considers successful—the patient living thirty-two days after operation.

F. T. Paul[8] proposes *gastrostomy in one stage* by the following operation: The preliminary stages are con-

A, Method of passing the ligatures in the stomach. 1, Glass tube. 2, Rubber tube with clip. 3, 3, Sectional View of glass rods over which the ligatures are tied. 4, Interior of the stomach. 5, Abdominal wall.

ducted as usual, but when the stomach is picked up a portion of it is drawn out of the wound, and two running sutures of fairly stout silk are passed in a circle round the site of the intended opening (A) with their ends in opposite directions, care being taken not to include the mucous membrane. The opening is then made, and each side of it being grasped with artery forceps, one of his small (three-eighths of an inch) intestinal glass drainage tubes is inserted and the ligatures are drawn tight and tied. The exposed part of the stomach is now washed and returned into the abdomen, the external wound drawn together with fishing gut sutures, and the ends of the stomach ligatures tied over two glass rods (3, 3) crossing the wound in order that the stomach may be kept in close contact with the peritoneal surface of the abdominal wall. Paul's experience of bowel cases has shown him that these tubes separate between the third and the seventh days; therefore until the third day the administration of food or washing out of the stomach may be carried out with impunity. On the morning of the third day the wound should be dressed, and from this time until the tube separates, and it is clear that good adhesions have formed, discretion in the administration of food should be exercised. He has operated on one case by this method and it answered well so far as the opening in the stomach was concerned.

**Gastro-enterostomy.**—For *cancer of pylorus* this does not appear to be attended with much success. Banks[9] reports two unsuccessful cases which were performed at the urgent request of the patient. He does not intend to perform this operation again in such cases, and taking it all round he is of opinion that if Billroth's celebrated case had not been successful, the average duration of life of patients suffering from pyloric cancer would have been prolonged.

**Cyst of the Stomach Wall.**—Ziegler relates[10] the following rare traumatic case. A man, aged twenty-three, received a severe injury in the region of the stomach as the consequence of a buffer accident. This was followed by hæmoptysis and hæmaturia. Abdomen was distended but no tumour could be felt. A fortnight later he had pain in the epigastrium, and a swelling could then be made out beneath the costal arch. Symptoms of intestinal obstruction appeared from time to time, but yielded to large enemata. Three-fourths of a litre of dark blood was drawn off from the swelling. The symptoms and tumour disappeared, but both returned in a few days. The swelling was felt between the umbilicus and left hypochondrium, and was elastic, fluctuating, and tender. It appeared to be immovable, and could not be marked off from the liver and spleen. The colon lay below it. When the stomach was inflated the swelling became more obvious. An exploratory puncture gave a blackish brown fluid, slightly alkaline, with red and white cells and granular bodies. It was impossible to make a differential diagnosis between an encapsuled blood effusion into the peritoneal cavity, rupture of the stomach or intestine, with consequent adhesions, pancreatic cyst, cyst of omentum or mesentery, or less probably a cyst of the liver, spleen, kidney, &c. Five months after the injury the abdomen was opened, when a large cyst covered with peritoneum presented. It occupied the anterior stomach wall from the pylorus until it was lost over the left end of the stomach. The omentum was inserted into its lower border. Three litres of fluid were withdrawn by a trocar. The swelling collapsed, and could not be moved on the stomach wall. A month later, when the patient was discharged, there was still a little resistance in the epigastrium. This disappeared later on. Two explanations are possible—(1) A blood effusion had taken place within the stomach wall, which produced the separation of its coats; or (2) an extravasation of lymph mixed with blood had occurred. The absence of clots, the character of the contents, the gradual appearance of the fluid, the re-collection after the first tapping, and the absence of any marks of injury on the sac wall were in favour of the latter view.

[1] Annals of Surgery, Jan., 1894. [2] Lancet, Feb. 17th, 1894, p. 412; and Brit. Med. Journ., Feb. 3rd, 1894, p. 243. [3] Amer. Journ. of the Med. Scien., Nov., 1893, p. 567. [4] Internat. Med. Mag., Jan., 1894, p. 1,124. [5] Annals of Surgery, Dec., 1893; and Amer. Journ. of Med. Scien., March, 1894. [6] Edinburgh Medical Journal, Feb., 1894, p. 708. [7] Arch. Prov. de Chir., 1893, tome ii., No. 5; and Amer. Journ. Med. Scien., March, 1894. [8] Lancet, Dec. 23rd, 1893, p. 1,562. [9] Lancet, Mar. 10th, 1894. [10] Münch. Med. Woch., Feb. 6th, 1894; and Brit. Med. Journ., March 3rd, 1894.

## Figures and Tables

**Figure f1:**